# A review study on the effect of Iranian herbal medicines against *in vitro* replication of herpes simplex virus 

**Published:** 2016

**Authors:** Mohammad-Taghi Moradi, Mahmoud Rafieian-Kopaei, Ali Karimi

**Affiliations:** 1 *Students Research Committee, Shahrekord University of Medical Science, Shahrekord, Iran*; 2 *Medical Plants Research Center, Shahrekord University of Medical Science, Shahrekord, Iran*

**Keywords:** *Herpes Simplex Virus*, *Herbal medicine*, *Iran In-vitro*

## Abstract

**Objective:**

There are a number of published data indicating *in vitro* anti-HSV activity of some of Iranian herbal extracts with no systematic review to discuss these results. Therefore, this article was aimed to review and discuss the methods carried out and the phytochemistry and bioactivity of the extracts used and also conclusions provided in these publications.

**Materials and Methods:**

Published articles both in English (from Medline, Science Direct, EMBASE, Scopus, Pro Quest, Google scholar, Cochrane Library) and in Persian (from SID, Iran Medex and Magiran) databases, from 1966 to October 2014 were incorporated in this review. The *in vitro* studies that lacked CC_50_, IC_50_, were excluded.

**Results:**

Only 42 published reports were found to examine Iranian herbs against HSV replication *in vitro. *Seventeen out of 42 studies in which 23 kinds of medicinal plants were subjected to crude extraction were included. The review of data showed that some of the herbal extracts including *Hyssopus officinalis *methanolic extract, *Melissa officinalis *aqueous extract, *Quercus persica *L. hydroalcoholic extract and *Securigeras ecuridaca *methanolic extract with selective index (SI) of 234, 877, >778 and 250, respectively were highly effective against HSV *in vitro.*

**Conclusion:**

More comprehensive studies using more advanced methods are needed to be done to achieve promising anti-HSV agents from the bioactive compounds isolated from these herbs.

## Introduction

Belonging to the family of Herpesviridae and subfamily of Alphaherpesvirinae, herpes simplex virus type-1 (HSV-1) and type 2 (HSV-2) are two of the most common human pathogens. Infections caused by these viruses are public health concern worldwide. These infections range from inapparent to severe life threatening infections such as encephalitis (Koelle and Corey, 2008 ▶). Although HSV-1 and HSV-2 are usually transmitted via different routes and infections caused by these two viruses involve different areas of the body, there is a great deal of overlapping features between the epidemiology and clinical manifestations of their infections. It is generally accepted that HSV is involved in oral diseases as HSV-1 is associated with recurrent aphthous ulceration (Malvy et al., 2007 ▶). HSV-1 infection commonly occurs in the mouth and lips resulting in gingivostomatitis (Malvy et al., 2007 ▶).

During the past two decades, the mechanisms of replication and pathogenesis of HSV-1 and thus the potential antiviral targets in this virus have been widely understood leading to development of antiviral compounds that target these mechanisms (Koelle and Corey, 2008 ▶). The antiviral drugs that are most commonly used to treat HSV-1 infections are nucleoside analogues such as Acyclovir (ACV) and Penciclovir of which Acyclovir is widely used as a drug of choice (Villarreal, 2003 ▶). However, a major problem associated with the use of ACV, is the development of drug-resistant HSV strains, particularly in AIDS patients (Elion, 1993 ▶). This kind of drug-resistance may occur after long-term treatment and is mainly due to mutations in HSV-1 thymidine kinase and/or DNA polymerase genes (Hill et al., 2009 ▶).

As potential alternatives to these agents, antiviral agents from medicinal plants with effective compounds exhibiting different modes of action against HSV infections are urgently needed. These natural active compounds are potential source of novel antiviral agents that could be widely used against these viruses. Some of these compounds have been identified to be active against HSV infections. They have high chemical diversity with biochemical specificity and have biologically active small molecules with drug-like properties that can be absorbed and metabolized in the body (Kitazato et al., 2007 ▶). Furthermore, they are easily available and no laborious pharmaceutical work is needed for their synthesis. Therefore, these natural products may be good candidates for development of special anti-HSV agents (Girish and Pradhan, 2008 ▶; Li et al., 2009 ▶). 

To the best of our knowledge, so far, no systematic review has been published regarding Iranian medicinal plants. Therefore, this article was aimed to review and summarize published results reporting Iranian medicinal plants with *in vitro* activity against HSV replication. This is to provide some recommendations for future research.

## Materials and Methods

To provide this systematic review, a number of databases including Medline, Science Direct, EMBASE, Scopus, Pro Quest, Google scholar, Cochrane Library Database and Persian databases such as SID, Iran Medex and Magiran, from 1966 to October 2014 were searched. The terms Herpes simplex virus, anti-viral, plant (herb), Iran, and natural or herbal medicine, were used as keywords. The databases used were limited to those Iranian articles published both in English and in Persian. In this review, i*n-vitro* studies lacking CC_50_, IC_50_, were excluded. All selected articles were studied by two reviewers to examine inclusion criteria and including common and scientific names of herbs, and their outcome. 

## Results

Using on line database search, only 42 published reports that examined Iranian herbs for *in vitro* inhibition of HSV replication were found*. *In this systematic review, 17 studies in which 23 kinds of medicinal plants were subjected to crude extraction were included and the remaining that used no standard methods to evaluate CC_50_, IC_50_, and SI, were excluded. The review of data showed that some of the herbal extracts including *Hyssopus officinalis *methanolic extract, *Melissa officinalis *aqueous extract, *Quercus persica *L. hydro alcoholic extract and *Securigeras ecuridaca *methanolic extract with SI of 234, 877, >778 and 250, respectively were highly effective against HSV, *in vitro* (Astani et al., 2014 ▶; Behbahani, 2009 ▶; Behbahani et al., 2013a ▶; Karimi et al., 2013 ▶). Data of these published studies are summarized in Table 1.

**Table 1:** *In vitro* anti-HSV characteristics of some of Iranian herbal medicines

**No**	**Botanical name**	**Extract**	**Common name**	**Parts**	**Family**	**Type of virus**	**IC**_50_ **(μg/ml)**	**CC**_50 _ **(μg/ml)**	**SI**	**references**
**1.**	*Aloe vera*	hot glycerine extract	Sabrezard	Leaf	Xanthorrhoeaceae	HSV-2	536	3238	7.56	(Zandi et al., 2007)
**2.**	*Avicenna marina*	methanol extract	*Harra*	Leaf	Avicenniaceae	HSV-2	10.5	262.5	25	(Behbahani et al., 2013b)
**3.**	*Avicenna marina*	Methanolic extract	*Harra*	Leaf	Avicenniaceae	HSV-1	80	>200	8.67	(Namazi et al., 2013)
**4.**	*Avicenna marina*	Ethanolic extract	*Harra*	Leaf	Avicenniaceae	HSV-1	192	200	0.17	(Namazi et al., 2013)
**5.**	*Avicenna marina*	Water extract	*Harra*	Leaf	Avicenniaceae	HSV-1	>200	>200	1.21	(Namazi et al., 2013)
**6.**	*Avicenna marina*	Chloroformic extract	*Harra*	Leaf	Avicenniaceae	HSV-1	180	110	0.001	(Namazi et al., 2013)
**7.**	*Avicenna marina*	n-hexanic extract	*Harra*	Leaf	Avicenniaceae	HSV-1	176	35	0.007	(Namazi et al., 2013)
**8.**	*Avicennia marina*	crude hot glycerine extract	*Harra*	Leaf	Avicenniaceae	HSV-1	137	5751	41.9	(Zandi et al., 2009)
**9.**	*Cuminumcyminum*	Methanolic extract	Zirehsabz	Seed	Apiaceae.	HSV-1	180	450	2.5	(Motamedifar et al., 2010)
**10.**	*Thymus kotschyanus*	aqueous extracts	Avishan	Flower+ leaf	Lamiaceae	HSV-1	100	800	8	(Farahani, 2013)
**11**	*Echinacea purpurea*	aqueous extracts	Sarkharghol	Root	Asteraceae or Compositae	HSV-1	500	900	1.8	(Farahani, 2013)
**12**	*Camellia sinensis*	aqueous extract	Chai	Leaf	Theaceae	HSV-1	50	1000	20	9Farahani, 2013)
**13**	*Echiumamoenum* L*.*	aqueous extract	Goleghavzaban	Flower	Boraginaceae	HSV-1	500	1000	2	(Farahani, 2013)
**14**	*Hyssopus officinalis*	Methanol96% extract	Zofa	Leaf	Lamiaceae	HSV-1	4.1	960	234	(Behbahani, 2009)
**15**	*Melissa officinalis*	aqueous extract	Badrangboyeh	Leaf	Lamiaceae	HSV-1	0.4	350	875	(Astani et al., 2014)
**16**	*Euphorbia spinidens*	methanolic extract	Shirmal	aerial parts	Euphorbiaceae	HSV-1	5072	320	15.85	(Mohammadi-Kamalabadi et al., 2014)
**17**	*Eucalyptus amigdalina*	hydroalcoholic extract	okaliptus	Leaf	Myrtaceae	HSV-1	180	650	3.6	(Karimi, 2012)
**18**	*Quercuspersica*	hydroalcoholic extract	Baloot	Seed	Fagaceae	HSV-1	257	>200000	>778	(Karimi et al., 2013)
**19**	*Myrtuscommunis*	hydroalcoholic extract	Moord	Leaf	Myrtaceae	HSV-1	3100	4960	1.6	(Moradi et al., 2011)
**20**	*Curcuma longa*	*-*	Zardchbeh	Root	Zingiberaceae	HSV-1	33.0	484.2	14.6	(Zandi et al., 2010)
**21**	*oleaeuropaea*	hydroalcoholic extract	zeyton	Leaf	Oleaceae	HSV-1	660	1750	2.6	(Motamedifar et al., 2007)
**22**	*Securigerasecuridaca*	Methanolic extract	Adasallmolok	Seed	Leguminosae	HSV-2	1.6	130	81.2	(Sayedipour et al., 2012)
**23**	*Securigerasecuridaca*	Methanolic extract	Adasallmolok	Seed	Leguminosae	HSV-1	2	500	250	(Behbahani et al., 2013a)
**24**	*Glycyrrhizaglabra*	*-*	Shirinbayan	Root	Leguminosae	HSV-1	500	800	1.6	(Monavari et al., 2008)
**25**	*Zataria Multiflora *Boiss	essential oil	Avishansirazi	Leaf	Lamiaceae	HSV-1	0.0059%	0.067%	11.7	(Mardani et al., 2012)

IC_50_: 50% inhibition of viral cytopathic, CC_50_: 50% cytotoxic concentration, SI: Selectivity index (CC_50_/IC_50_), HSV-1: herpes simplex virus type 1, HSV-2: herpes simplex virus type 2

Since each extract with SI≥4 should be considered as a potential antiviral agent (Tsuchiya et al., 1985), the characteristics of some of the extracts isolated from some Iranian medicinal plants are discussed below.

***Avicenna marina***

*Avicenna marina* (commonly known as grey mangrove or white mangrove), is one of the mangrove species plants which is native to Southern Africa (Duke, 1991 ▶). This plant grows in Hara forests of Persian Gulf and is used in Persian folk medicine. Leaf extracts of *A. marina* contain compounds such as iridoid glucosides, fatty acids, sterols and hydrocarbons (Hogg and Gillan, 1984 ▶; König and Rimpler, 1985 ▶). 

Based on the results of a study, the methanol extract of this herbal medicine showed higher antiviral activity against HSV, *in vitro*. The most polar fraction (D) of the methanol extract showed significant anti-HSV activity. This fraction inhibited the HSV replication following entry of the virus into the target cells. The phytochemical examinations of this extract have shown that fraction D contains flavonoid compounds. It has been shown that *A. marina* contains active flavonoid compounds which inhibit the replication of HSV (KOS strain) following penetrating into the target cells (Namazi et al., 2013 ▶). 

Behbahani et al showed that crude methanol extract of *A. marina* and the corresponding fractions LMEG and luteolin inhibit HSV-2 replication with selective index (SI) of 25, 25 and 5.3, respectively. The results indicated that LMEG was the most active sub-fraction of *A. marina* with inhibitory effect on the early stage of HSV-2 replication (Behbahani et al., 2013b ▶). Zandi et al showed that crude hot glycerin extract of fresh leaves of *A. marina* has antiviral activity against HSV-1 replication, *in vitro*, mostly before adsorption of the virus to the cells (Zandi et al., 2009 ▶).

***Aloe vera***

*Aloe vera* is a member of the Liliaceae family. Historically, it has been used for medical purposes such as treatment of wounds and burns (Perfect et al., 2005 ▶). This medicinal plant is the source of two herbal preparations namely, aloe gel and aloe latex. Aloe gel is often referred to a clear gel or mucilaginous substance produced by parenchymal cells located in the central region of its leaves. Diluted aloe gel is commonly referred to as *A. vera* extract. The main components of this gel are water (99%), monosaccharides, and polysaccharides (25% of the dry weight of the gel) (Shelton, 1991 ▶). There are some published reports indicating that the extracts of *A. vera *and particularly its anthraquinone compounds have antiviral activity (Andersen et al., 1991 ▶; Sydiskis et al., 1991 ▶). It has been shown that crude hot glycerine extract of *Aloe vera* gel, grown in southwest of Iran, Bushehr, inhibits HSV-2 replication, both before and after attachment to the Vero cell line (Zandi et al., 2007 ▶). 

***Hyssopus officinalis***

*Hyssopus officinalis* is a member of the Lamiaceae family. Some members of this family such as *Origanum vulgare* are known to be poisonous and traditionally used as an arrow poison. Growing in different geographical regions of Iran, *H. officinalis*, mainly its leaves, has been used in the traditional Iranian medicine (TIM) for treatment of various ailments (Kazazi et al., 2007 ▶). Methanolic extract of the leaves of *H. officinalis*, has exhibited remarkable activity against both wild type and resistant strains of HSV, *in vitro*. The extract, also, significantly inhibited plaque formation of both of the two strains of HSV-1 in Vero E6 cells with minimal cell cytotoxicity (CC_50_ = 960 μg/ml). An oral dose of the extract (125 mg/kg) significantly delayed the onset of HSV-1 infection by over 50%. It also increased the mean survival time of infected mice by 55-65% compared to the non-treated mice (p<0.05). The mortality rate for mice treated with the extract was also significantly reduced by 90% in comparison with the non-treated mice that exhibited 100% mortality (Behbahani, 2009 ▶). 

***Melissa officinalis***

Leaves of *Melissa officinalis* (lemon balm), a member of the Lamiaceae family (Saller et al., 2001 ▶; Wolbling and Leonhardt, 1994 ▶) contain polyphenols such as rosmarinic acid and flavonoids (Carnat et al., 1998 ▶). *M. officinalis* has been evaluated for its activity against HSV, *in vitro* (Geuenich et al., 2008 ▶; Nolkemper et al., 2006 ▶). Aqueous extract of *M. officinalis* has been shown to interact directly with free viral particles of two acyclovir-resistant and one acyclovir-sensitive strains of HSV-1 at low IC_50_ values of 0.13, 0.23 and 0.4 μg/ml and high selectivity indices of 2692, 1522 and 875, respectively with inhibiting attachment of these strains to the host cells in a dose-dependent manner. The results of this work indicated that anti-HSV-1 activity of Melissa extract was mainly due to its rosmarinic acid contents (Astani et al., 2014 ▶). 

***Zataria multiflora***

*Zataria multiflora* Boiss (Avishan-e-Shirazi in Persian and Sa'atar or Zaatar in ancient Iranian medical books) is a thyme-like plant and a member of Lamiaceae family that wildly grows in central and southern parts of Iran (Amin, 1991 ▶). It is used in traditional folk remedies for its antiseptic, analgesic (pain-relieving) and carminative (anti-flatulence and intestine soothing) properties (Amin, 1991 ▶; Mozaffarian, 1996 ▶). In addition to antibacterial activity (Eftekhar et al., 2011 ▶), essential oil of *Zataria multiflora *Boiss has been shown to have significant inhibitory effect on HSV-1 replication with IC_50_ values and selectivity indices of 0.0059% and 11.7, respectively (Mardani et al., 2012 ▶).

***Quercus persica***

*Q. persica *“oak” is a tree of which about 600 species exist around the world. *Quercus* species contain high levels of tannins in both hydrolysable and condensed (Rakić et al., 2007 ▶) forms. Oak fruit (acorn) comprises starch, protein, oil, and tannins with high amounts of amylose, amylopectin, high molecular weight and viscosed substances (Saffarzadeh et al., 1999 ▶). This fruit is useful in treatment of anemia, diarrhea and is used in livestock feeding. There are some published reports indicating antiviral (Karimi et al., 2013 ▶; Muliawan et al., 2006 ▶), antibacterial (Khosravi and Behzadi, 2006 ▶), and anti-inflammatory (Kaur et al., 2004 ▶) potentials of *Quercus *species. These effects have been attributed to its phenolic contents, particularly to its flavonoid and tannin compounds (Chang et al., 2002 ▶). It has been suggested that tannin-based components of oat fruits have remarkable effect on virus replication with different mechanisms and with the minimum cytotoxicity (Buzzini et al., 2008 ▶). In our previous study, *Q. persica* L. had highly anti-HSV-1 activity and very low cytotoxicity *in vitro*, as exhibited no cytotoxic effect on this cell line up to concentration of 200 mg/ml. Using inhibition assay, this extract inhibited HSV-1 replication by reduction of its cytopathic effect (CPE) before and after cellular attachment in concentrations of 1.02 and 0.257mg/ml, respectively (Karimi et al., 2013 ▶). 

***Thymus kotschyanus***

Lamiaceae (formerly Labiatae) is one of the most important plant families in which *Thymus* with about 215 species, is a significant genus (Zaidi and Crow, 2005 ▶). The plants of this genus are well known as medicinal plants because of their biological and pharmacological properties. Fourteen species of this genus are represented in Iranian flora, of which *T. daenensis *Celak, *T. kotschyanus *Boiss, and *T. Hohen* are widely used as medicinal plants. Infusion and decoction of aerial parts of these species are used in Iranian traditional medicine (ITM) as tonic, carminative, digestive, antispasmodic, anti-inflammatory, anti-tussive and expectorant and for the treatment of common colds (Zargari, 1990 ▶). Aqueous extract of *T. kotschyanus* has been shown to inhibit HSV-1 *in vitro* with IC_50_ value and selectivity indices of 100μg/ml and 8, respectively (Farahani, 2013 ▶).

***Securigeras ecuridaca***

*Securigeras ecuridaca *(family Fabaceae), also called goat pea, is an annual herb used against a variety of diseases such as malaria, gastric influx, epilepsy, hypertension and hyperlipidemia in eastern folk medicine (Garjani et al., 2009 ▶; Pouramir et al., 2011 ▶). Compounds such as cardenolides, steroidal and pentacyclic have been detected in extract of *S. securidaca* seeds (Zatula et al., 1969 ▶). Kaempferol and kaempferol-7-O-glucoside have also been isolated from *S. securidaca* with potential activity against HSV-1 and HSV-2 replication (Behbahani et al., 2013a ▶; Sayedipour et al., 2012 ▶). Behbahani et al, evaluated anti HSV-1 activity of 20 µg/ml of both crude methanol extract and twelve associated fractions of *S. securidaca*. Their results showed that IC_50_ values of crude methanol extract, kaempferol, kaempferol-7-O glycoside and Acyclovire (ACV) were 2±0.12, 0.2±0.01, 0.1±0.01 and 0.1±0.01 μg/ml, respectively. Also, the calculated selective index (SI) for crude methanol extract, kaempferol and kaempferol-7-O-glycoside were 250, 300 and 2500, respectively (Behbahani et al., 2013a ▶). It has also been shown that crude methanol extract of *S. securidaca* had inhibitory effect on HSV-2 in cell culture with IC_50_ values and selectivity indices of 1.6 μg/ml and 81.2, respectively (Sayedipour et al., 2012 ▶).

***Curcuma longa***

*Curcuma longa *L. has been used in Asian traditional medicine (ATM) for a long time to treat gastrointestinal upset, arthritic pain, urinary tract infection, and liver ailments (Dixit et al., 1988 ▶; Luper, 1999 ▶). One of its main component, Thcurcumin has been shown to have several pharmacological activities such as anti-inflammatory, anti-oxidant, anti-microbial properties (Maheshwari et al., 2006 ▶), and antiviral activity against hepatitis B virus (HBV) by decreasing the transcription of HBV X (HBx) gene (Kim et al., 2009 ▶). Antiviral activity of different concentrations of curcumin, gallium-curcumin and Cu-curcumin were tested against HSV-1 and the results showed that curcumin and its new derivatives had remarkable antiviral effects on replication of this virus in cell culture. The IC_50_ values of curcumin, gallium-curcumin and Cu-curcumin were 33.0, 13.9 and 23.1 µg/ml, respectively and their calculated SI values were 14.6, 18.4 and 14.1, respectively (Zandi et al., 2010 ▶). 

***Camellia sinensis***

Tea is a cultivated evergreen plant and its green, oolong and black types are all made from the same plant species, *Camellia sinensis *L. Their differences in appearance, organoleptic taste, and chemical content as well as flavour are due to their fermentation process. The chemical components of its leaves include polyphenols (catechins and flavonoides), alkaloids (caffeine, theobromine, the ophylline, etc.), volatile oils, polysaccharides, amino acids, lipids, vitamins (e.g., vitamin C), inorganic elements (e.g., aluminium, fluorine and manganese), etc. The beneficial properties of tea are attributed to its polyphenols. Its flavonoides have antioxidant, anti-inflammatory, anti-allergic and anti-microbial effects. The polyphenols content of green tea and black tea varies from 30% to 40% and 3% to 10%, respectively. Tea and particularly, green tea has been evaluated for its medicinal properties and a number of studies suggested that chemical constituents of tea play important role in human health (Sharangi, 2009 ▶). It was shown that *in vitro* replication of HSV-1 is inhibited by aqueous extract of *C. sinensis* with IC_50_ value and selectivity index of 50 and 20 μg/mL, respectively (Farahani, 2013 ▶).

***Euphorbia spinidens***

Plants in the family of Euphorbiaceae are well known for the chemical diversity of their isoprenoid constituents (Jury et al., 1987 ▶). Members of the *Euphorbia* genus are known to contain substances with inhibitory action against some bacteria. This inhibitory action has been attributed to their large amounts of phenolic compounds (Li et al., 2009 ▶). *E. spinidens* is an indigenous plant naturally growing in different geographical regions of Iran and is increasingly used in traditional medicine. Crude methanol extract of *E. spinidens* and the corresponding fractions n-Hexane, Chloroform, and n-Butanol, with SI values of 15.85, 2.062, 8.2, and 25.37, respectively, inhibited early stages of HSV-1 replication, *in vitro* (Mohammadi-Kamalabadi et al., 2014 ▶).

## Conclusion

Searching for new bioactive compounds in order to develop new anti-viral agents seems to be urgent. Research on some medicinal plants may potentially lead to isolation of active compounds which could be effective against HSV. To achieve this goal, both experimental and clinical investigations are needed to provide a better understanding of mechanisms of action, therapeutic effects, and the safety profile of these compounds. Iran with a diverse climate and wide variety of herbal flora could be considered as potentially a great source of these herbs from which isolation of these components is possible. Therefore, this systematic review which covered the published results of research on some Iranian medicinal plants and the corresponding isolated active phytochemicals, conclusively, would highlight the need for more comprehensive studies using more advanced methodology to develop promising anti-HSV agents from these bioactive compounds.

## Note

*Please cite this paper as: *

Moradi MT, Rafieian-Kopaei M, Karimi A. A review study on the effect of Iranian herbal medicines against *in vitro* replication of herpes simplex virus. Avicenna J Phytomed, 2016; 6 (5): 506-515.
